# Succession and Diversity of Microbial Flora during the Fermentation of Douchi and Their Effects on the Formation of Characteristic Aroma

**DOI:** 10.3390/foods12020329

**Published:** 2023-01-10

**Authors:** Huiyan Zhao, Jingting Xu, Ruican Wang, Xinran Liu, Xingyun Peng, Shuntang Guo

**Affiliations:** 1 Beijing Key Laboratory of Plant Protein and Cereal Processing, College of Food Science and Nutritional Engineering, China Agricultural University, Beijing 100083, China; 2 Tianjin Key Laboratory of Food Science and Health, School of Medicine, Nankai University, Tianjin 300071, China

**Keywords:** Douchi fermentation, dominant microorganisms, microbial diversity, volatile compounds, correlation analysis

## Abstract

This study aims to understand the development and succession of the microbial community during the production of traditional *Aspergillus*-type Douchi as well as their effects on the formation and variation of characteristic aroma compounds. High-throughput sequencing technology, solid-phase microextraction, gas chromatography–mass spectrometry, and Spearman correlation analysis were conducted to study the changes in the microbial community and characteristic flavor during the fermentation process. *Aspergillus* spp. was dominant in the early stage of fermentation, whereas *Staphylococcus* spp., *Bacillus* spp., and *Millerozyma* spp. became dominant later. At the early stage, the main flavor compounds were characteristic soy-derived alcohols and aldehydes, mainly 1-hexanol, 1-octen-3-ol, and nonanal. In the later stage, phenol, 2-methoxy-, and 3-octanone were formed. Correlation analysis showed that six bacterial genera and nine fungal genera were significantly correlated with the main volatile components, with higher correlation coefficients, occurring on fungi rather than bacteria. Alcohols and aldehydes were highly correlated with the relative abundance of bacteria, while that of yeast species such as *Millerozyma* spp., *Kodamaea* spp., and *Candida* spp. was positively correlated with decanal, 3-octanol, 2-methoxy-phenol, 4-ethyl-phenol, 3-octanone, and phenol. The novelty of this work lies in the molds that were dominant in the pre-fermentation stage, whereas the yeasts increased rapidly in the post-fermentation stage. This change was also an important reason for the formation of the special flavor of Douchi. Correlation analysis of fungi and flavor substances was more relevant than that of bacteria. As a foundation of our future focus, this work will potentially lead to improved quality of Douchi and shortening the production cycle by enriching the abundance of key microbes.

## 1. Introduction

Douchi is a featured Chinese traditional fermented soybean condiment with good taste and distinct flavor, which is widely used as a condiment in Chinese dishes [1], such as fried fish, meat, vegetables, and canned foods. Based on the type of microorganisms involved, Douchi is often divided into four categories, i.e., *Aspergillus*-type, *Bacterial*-type, *Mucor*-type, and *Rhizopus*-type. As a typical seasoning Douchi, the *Aspergillus*-type Douchi stands out for its widespread popularity and high yields in the industry [2].

Numerous studies have confirmed that the unique flavor and taste of Douchi are closely correlated with the microbial flora present throughout the fermenting process. Yang et al. [3] studied the microbial diversity of *Aspergillus* Douchi and revealed that the primary bacteria were *Staphylococcus* spp. and *Welchiella* spp., while the main fungi were *Aspergillus* spp. and *Lichtheimer* spp. Yang et al. [4] analyzed the *Aspergillus*-type Douchi from Jiangxi, China, and reported the correlation between the relative abundance of *Weisseria* spp., *Bacillus* spp., anaerobic bacteria, *Lactobacillus* spp., *Enterococcus* spp., and *Staphylococcus* spp. and 58 dominant flavor substances. According to this study, *Lactococcus* spp., *Gulosibacter* spp., *Atopostipes* spp., *Corynebacterium_1*, *Peptostreptococcus* spp., and *norank_f__Actinomycetaceae* are the primary flavor-related bacteria that are functionally important in the Douchi fermentation process. The microbial community and fermentation metabolites of traditional fermented food will change dynamically during the fermentation process. Other studies have also revealed that the fermentation and metabolism of microorganisms, such as *Aspergillus* spp., *Lecanicillium* spp., *Staphylococcus* spp., *Pediococcus* spp., *Meyerozyma* spp., and *Candida* spp., are closely associated with the flavor of Douchi [5,6,7,8]. Many studies have shown that many metabolites are in the fermentation process, such as organic acids, amino acids, volatile compounds, and other metabolites [9]. It is reported that some Staphylococcus can produce 3-methyl butyric acid with cheese aroma through metabolism [10], and flavoring yeast plays an important role in the metabolism of soy sauce fermentation and the generation of flavor compounds [11]. Due to the diversity and complexity of microorganisms, metabolites, and flavors in the fermentation process, it is necessary to analyze the microbial community and flavor compounds in the whole fermentation period in stages to understand the fermentation characteristics of traditional fermented food.

Traditionally, the production of Douchi involves natural fermentation for a long period of time. Among them, Guangdong Yangjiang Douchi is an important category, with an annual sales volume of about 12,000 tons, accounting for 40% of the national market share, and playing an essential role in the development of China’s Douchi industry. The natural fermentation of Yangjiang Douchi from Guangdong, China, involves five stages, i.e., raw material pretreatment, pre-fermentation (koji making), koji washing, post-fermentation, and end of fermentation, which last for more than 35 days [12]. Such complex and time-consuming procedures are associated with various industrial issues, such as unmanageable environmental conditions and microbial growth patterns, resulting in low production efficiency, uncontrollable flavors, and batch-to-batch quality variation [13]. Although microorganisms have been known to be relevant to the flavor compounds in Douchi, we have limited knowledge about the succession of microbial communities and dynamic changes of fermentation substrates. Additionally, development of volatile characteristics at different fermentation stages remains unclear.

Therefore, the purpose of this study was to determine the involvement of core functional microorganisms in the formation of characteristic volatiles for different fermentation stages. To describe the microbial community succession during the five fermentation stages of Douchi, high-throughput sequencing was performed, and headspace solid-phase microextraction (HS-SPME) combined with gas chromatography–mass spectrometry (GC-MS) was used to evaluate the volatile compounds of Douchi. By way of chemical and statistical analyses, this work provides theoretical guidance to better understand and control the Douchi fermentation process.

## 2. Materials and Methods

### 2.1. Collection of Douchi Samples

**Figure 1:**
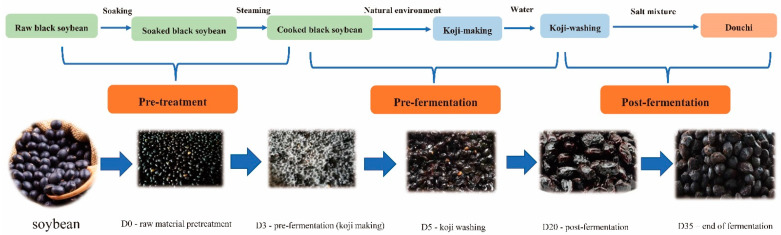
Sample diagram of Douchi in different fermentation stages.

Douchi samples were collected at a well-known traditional Douchi processing plant (Yang Fan Food Co., Ltd., Yangjiang, China). As shown in Figure 1, Douchi was prepared through natural fermentation, and the whole process was divided into two stages: (1) pre-fermentation (Koji-Making): samples were (F0, F3 and F5) collected on days 0 (beginning), 3 (koji-making), and 5 (koji-washing), respectively; and (2) post-fermentation: samples (F20 and F35) were collected on days 20 (aging) and 35 (finishing), respectively. On each sampling day, 3 aliquots (500 g) of samples were collected from 3 different fermenters, combing samples from the upper, medium, and lower levels of a fermenter. Afterwards, the three aliquots were mixed up in a pail. All samples were labeled and maintained in plastic sterile bags at −80 °C until further examination.

### 2.2. Extraction and Sequencing of DNA

#### 2.2.1. DNA Extraction from Bacteria and Fungi

From Douchi samples, bacterial and fungal DNAs were extracted using the FastDNA^®^ Spin Kit for soil (MP Biomedicals, Santa Ana, CA, USA) according to the manufacturer’s instructions. The quality of the extracted DNA was analyzed by separating it on a 1% agarose gel electrophoresis and determining its concentration and purity with a NanoDrop 2000 UV–vis spectrophotometer (Thermo Scientific, Wilmington, NC, USA). The DNA was frozen at −80 °C for further analysis.

#### 2.2.2. Amplification and Sequencing by Illumina MiSeq

The primers 338F (5′-ACTCCTACGGGAGGCAGCAG-3′) and 806R (5′-GGACTACHVGGGTWTCTAAT-3′) were designed based on the V3–V4 hypervariable regions of the bacteria 16S rRNA gene, and the ITS1F region of fungi was amplified with the forward primer (5′-CTTGGTCATTTAGAGGAAGTAA-3′) and the reverse primer ITS2R (5′-GCTGCGTTCTTCATCGATGC-3′) using a thermocycler polymerase chain reaction (PCR) system (GeneAmp 9700, ABI, Houston, TX, USA), respectively.

The following program was used for the PCR reactions: 3 min of denaturation at 95 °C, followed by 27 cycles of 30 s at 95 °C in 338F_806R (35 cycles of 30 s at 95 °C in ITS1F_ITS2R), annealing at 55 °C for 30 s, and extension at 72 °C for 45 s, and single extension at 72 °C for 10 min. Each PCR reaction contains 4 μL of 5 × TransStart FastPfu Buffer, 2 μL of 2.5 mM dNTPs, 0.8 μL of forward and reverse primers (both at 5 μM), 0.4 μL of TransStart FastPfu DNA Polymerase, 10 ng of template DNA, and up to 20 μL of ddH_2_O. Triplicate PCR reactions were run. Using the AxyPrep DNA Gel Extraction Kit (Axygen Biosciences, Union City, CA, USA), we purified the PCR product from a 2% agarose gel and then used a QuantiFluor™ -ST (Promega, Madison, WI, USA) to determine its quantity.

Lastly, the libraries of bacterial 16S rRNA genes and fungal ITS rRNA genes were sequenced using an Illumina MiSeq platform (Illumina, San Diego, CA, USA), with the assistance of Majorbio Bio-Pharm Technology Co., Ltd. (Shanghai, China) for library preparation and sequencing.

#### 2.2.3. Bioinformatics Analysis

Trimmomatic filtered the quality of raw fastq files and FLASH (version 1.2.11) merged them. Using the Silva database (http://www.arb-silva.de, accessed on 22 July 2022) and the Unite database (version 7.0 http://unite.ut.ee/index.php, accessed on 10 August 2022), a Qiime platform (http://qiime.org/install/index.html, accessed on 10 August 2022) RDP classifier (version 2.2 https://sourceforge.net/projects/rdp-classifier/, accessed on 15 August 2022) was used to analyze the taxonomy of raw sequencing reads with a confidence threshold of 70%. Usearch (version 7 http://www.drive5.com/usearch/, accessed on 26 August 2022) was used to cluster operational taxonomic units (OTUs) based on their similarity to one another, with a threshold of 97%. Clustering and multi-analysis were performed using the i-Sanger platform built by Majorbio Bio-Pharm Technology Co., Ltd.

### 2.3. Analysis of Volatile Compounds in Douchi

#### 2.3.1. Isolation of Volatile Compounds from Traditional Fermented Douchi

The volatile compounds from fermented Douchi were extracted using headspace solid-phase microextraction (HS-SPME) according to Peng et al. [14], and appropriate modifications were made. A 15 mL headspace vial was filled with a 1.5 g Douchi sample, 1.25 g of sodium chloride, and 5 mL of distilled water. In all, 10 μL of 2-Octanol diluted 1 × 10^4^ times in methanol served as an internal standard. Vial samples were pre-equilibrated in a 50 °C water bath (DK-S24, Shanghai, China) for 20 min. To extract the volatile compounds, an aged 50/30 μm DVB/CAR/PDMS (2 cm) SPME fiber (Supelco, Bellefonte, PA, USA) was inserted into the headspace vial and kept at 0.5–1 cm from the sample for 40 min. The fiber was retracted after extraction and then inserted into the GC-MS system’s injection port, where it was heated to 250 °C for 5 min. Each sample was analyzed for three times.

#### 2.3.2. Using Gas Chromatography–Mass Spectrometry to Identify and Quantify Volatile Compounds 

All samples’ volatile components were analyzed using an Agilent 7890B gas chromatograph system and an Agilent 5977B mass spectrometer (MSD) (Agilent Technologies, Santa Clara, CA, USA) equipped with a DB-WAX capillary column (30 m × 0.25 mm × 0.25 μm film thickness). The carrier gas for the column was high-pure helium with a purity of 99.999%, and the flow rate was 1.0 mL/min. The gas chromatograph’s injection mode was set to splitless, and the injector temperature was 250 °C. The GC oven was held to 40 °C for 3 min, then raised to 130 °C at a rate of 3 °C/min and held for 5 min, before being raised to 200 °C at a rate of 4 °C/min and kept for another 5 min. The following parameters were used for MS: a mass spectrometer having a mass range of 35–500 m/z; 70 eV electron ionization; 230 °C ion source and 150 °C quadrupole temperature. Using the retention times of a series of C6–C20 n-alkane, the linear retention indices of all identified compounds and reference standards were calculated. 

### 2.4. Statistical Analysis

Data were provided as mean ± standard deviation, and all studies were performed in triplicate. TBtools [15] and SPSS software (version 26.0, Inc. Chicago, Chicago, IL, USA) were used to generate a heatmap and perform correlation analysis, respectively. SIMCA-P software (version 14.1, Umetrics, Ume, Sweden) was used for principal component analysis (PCA). GraphPad Prism (version 9, GraphPad Software, San Diego, CA, USA) and the Majorbio Bio-Pharm plotting platform were used for data visualization. The OmicStudio tools (https://www.omicstudio.cn/tool, accessed on 10 September 2022) were used to create the correlation network.

## 3. Results and Discussion

### 3.1. Yangfan Douchi Microbial Diversity during Different Stages of Fermentation

Traditionally, the production of Douchi involves a natural fermentation process where cooked soybean seeds are placed in a room with relatively stable temperature and humidity for a period (at least 35 days). To better understand the characteristics and dynamic succession of the microbial community throughout processing, this research utilized Illumina Miseq high-throughput sequencing technology to analyze the alpha diversity of the microbial community in Yangjiang Douchi samples during different fermentation stages. The original data obtained from sequencing were processed for quality control. Results showed that a total of 881,183 effective fungal sequences were recovered from 15 Douchi samples, with a read length of 260.43 bp on average. In addition, 935,870 valid bacterial sequences were identified, with a read length of 415.82 bp on average. After truncating the sequences at 97% sequence similarity level for clustering, it was found that the total number of OTUs of fungi and bacteria were 216 and 1049, respectively. Clearly, in this study, bacterial diversity was found to be greater in the Yangjiang Douchi samples than fungal diversity.

**Figure 2:**
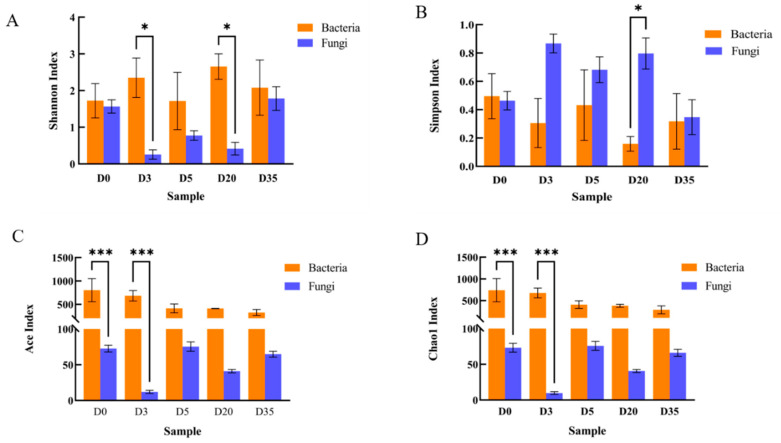
Alpha diversity of Douchi in different fermentation stages, (**A**): Shannon Index, (**B**): Simpson Index, (**C**): Ace Index, (**D**): Chao 1 Index (***: *p* < 0.001, *: *p* < 0.05).

Shannon index and Simpson index were both good indicators of the variety of microbial communities. Shannon’s index increases with species richness, while Simpson’s index decreases in that direction [16,17]. As shown in Figure 2A, during the fermentation of Douchi, the variation range of bacteria’s Shannon index was significantly greater than that of fungi. A minimal Shannon Index difference was noted between bacteria and fungi at the beginning of fermentation (D0), but it increased significantly at the koji-making stage (D3), decreased slightly after koji washing (D5), and recovered to or even exceeded the level of the koji-making stage (D3) at the late stage of fermentation (D20). Finally, we observed an overall increasing trend (D35) (Figure 2A). The Simpson index of bacteria exhibited the predicted reverse trend (Figure 2B). The fungal Shannon index decreased significantly after washing, followed by a late-on recovery to a level that was comparable to that of bacteria. In addition, the variation in the Simpson index corresponded to that in the Shannon index (Figure 2B). The above results demonstrated that the relative abundance and proliferation of both bacteria and fungi were affected by the Douchi processing conditions and showed some degree of fluctuation throughout the fermentation process. However, analyzing the whole fermentation process revealed that the results of diversity indices were consistent with the a forementioned OTU results, confirming that the bacterial diversity of Yangjiang Douchi was greater than the fungal diversity. The microbial community alpha diversity analyses managed to cover 99.99% of the specimens over different fermentation stages (Supplementary Table S1), indicating that the high sequencing quality was sufficient to cover the majority of the microorganisms in the samples. The results accurately represented the microbial community diversity in this sample. 

The Ace and Chao 1 indices are known to positively correlate with species richness and might thus be used to estimate microbial community richness. As shown in Figure 2C,D, the Ace and Chao 1 indices of bacteria were much higher than those of fungi in different periods of Douchi fermentation, and they showed the same patterns of variation, showing that the bacterial flora was far richer than the fungal flora. Bacterial Ace and Chao1 indices were significantly higher in the initial stage of Douchi which declined on day 5 (D5 sample) and remained stable thereafter. The Ace index and Chao 1 index of fungi decreased significantly after washing (D3), but it recovered significantly on day 5 (D5). There was a significant fluctuation in richness among D5, D20, and D35 samples, but the final Ace and Chao 1 indices (D35) were not noticeably distinct from early stages. These data indicated the presence of numerous bacteria and fungi species during the initial stage of Douchi fermentation; furthermore, due to the washing and fermentation processes, certain species formed the dominant flora while some species diminished, resulting in somewhat declined diversity. In general, the bacteria community showed greater diversity and richness than fungi.

### 3.2. Changes in Bacterial Community Diversity and Composition in Aspergillus-Type Douchi

**Figure 3:**
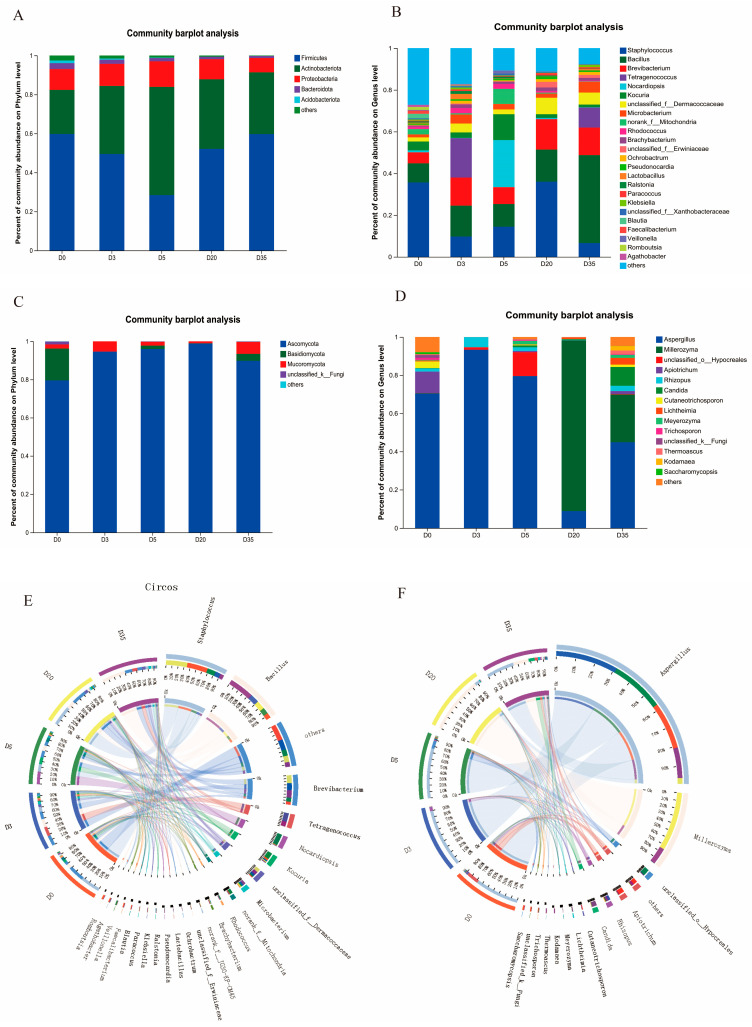
Characteristics of relative abundance of bacteria at the phylum (**A**) and genus (**B**) levels of *Aspergillus*-type Douchi from different fermentation periods. The relative abundance of fungi at the phylum (**C**) and genus (**D**) levels of *Aspergillus*-type Douchi during fermentation. Distribution of microbial community for each sample at the genus level. The data were visualized by Circos. The width of the bars from each genus indicates the relative abundance of that genus in the sample. (**E**): bacteria; (**F**): fungi.

To further clarify the succession of bacterial species during the fermentation of Douchi, bacterial composition was investigated at the phylum and genus levels, and the results are shown in Figure 3A. At the level of the phylum, Firmicutes, Actinobacteriota, and Proteobacteria were the main phyla throughout the fermentation process (D0 to D35, 15 samples in total). Among these dominant phyla, the relative abundance of Firmicutes remained over 50% on all stages except for D5 (decreased to 28.39%), which was considered the most active and abundant. The relative abundance of Actinobacteriota was the second highest of all time except for day 5, showing an opposite trend of variation compared to Firmicutes: it continuously increased in the early stages, reaching the highest (55%) on day 5, followed by a decrease to 31.68% in the late stages. The relative abundance of Proteobacteria, on the other hand, remained relatively low in both the early (10.69%) and late (10.56%) stages of fermentation. Bacteroidota and Acidobacteriota maintained extremely low abundance, with that of the post-fermentation stage lower than less than 1%. On the level of the genus, the results of the most abundant 15 strains are shown in Figure 3B. Bacteria that showed a high relative abundance included *Staphylococcus*, *Bacillus*, *Brevibacterium*, *Tetragenococcus*, and *Kocuria*. *Staphylococcus* and *Bacillus* were the predominant microorganisms during all phases of fermentation.

Circos visualized the bacterial community dispersion at the genus level, shown in Figure 3E. Within the whole fermentation system, *Staphylococcus* showed high abundance and stability. In the initial stage (D0) and the late stage (D20), its relative abundance was around 40%, which dropped to 6.62% in D35. These results suggested that a large amount of *Staphylococcus* was involved in the fermentation of Douchi, which may have a major influence on the formation of the characteristic flavor of Yangjiang Douchi.

*Bacillus* is a common and important fermenting bacterial genus in fermented soybean foods (e.g., natto and soybean paste) [18,19]. In this study, *Bacillus* showed relatively low abundance (9.16%) at the early stage of Douchi fermentation. However, it increased sharply at the late stage of fermentation, reaching 42.12% on D35, and took over the leading place of *Staphylococcus*. The analysis results were consistent with those in a previous study [20]. *Bacillus* had an absolute advantage in the post-fermentation process and could accelerate the fermentation of Douchi. On the other hand, *Tetragenococcus* was detected in D3 and D35 samples, and the abundance of *Tetragenococcus* reached 9.61% after 35-day fermentation. *Tetragenococcus* is widely found in traditional fermented foods, including soy sauce, fermented fish sauce, and cheese [21,22,23], and it has been proved to enhance the flavor of condiments, such as fish sauce. It can be deduced that *Tetragenococcus* is also a bacteria genre that cannot be ignored in the formation of Douchi flavor.

It could be seen from the above results that the bacterial community showed rich diversity throughout the fermentation of Douchi, and the relative abundance of bacteria changed dynamically along with the fermentation process. *Staphylococcus* was predominant with high abundance throughout the initial and intermediate stages of fermentation. *Bacillus* was low in the beginning stage of fermentation; however, it increased and peaked in the late stages, becoming the dominant bacteria in the community. *Tetragenococcus* existed at the early and middle stages of fermentation, and its role in the formation of Douchi flavor was also worthy of attention.

### 3.3. Changes in Fungal Community Diversity and Composition in Aspergillus-Type Douchi

Figure 3C,D show the variations in fungal composition at the levels of phylum and genus in Douchi fermentation. Figure 3C shows that four phyla, Ascomycota, Basidiomycota, Mucoromycota, and other unclassifiable fungi (those with abundance less than 1% were combined into the “others” category), were identified throughout the whole fermentation process. Ascomycota were prominent throughout the whole fermentation process of Douchi (Figure 3C). Its relative abundance was close to 80% in the early stage of fermentation (F0), increased steadily thereafter, and maintained a high level (F3 to F20, 94.61–98.84%) in the post-fermentation stages. The final abundance of Ascomycota was 90% on day 35 (F35). These results were similar to those reported to other Douchi produces (Liuyang Douchi) [24]. *Basidiomycota* was not a dominant fungi phylum, which showed an initial abundance of 16.65% and decreased to 3.63% at the end of fermentation. The third group was Mucoromycota, whose abundance was only 2.21% at the beginning. Although it did not occupy a dominant position after the beginning of fermentation, it showed an increasing trend until the end of fermentation (6.32%). Mucoromycota is the main fermentation bacteria of cheese and sufu [25], which can produce a great amount of proteases and lipases to act on protein and fat, producing rich flavor such as umami. Although the abundance of Mucoromycota was low in Douchi, its roles in flavor formation might be as important as those of the more abundant Basidiomycetes and Ascomycetes.

Figure 3D shows the top 15 fungi genera in Douchi, mainly molds and yeasts, and Circos’ visualization of genus-level community distribution data is shown in Figure 3F. The results demonstrated that molds were present throughout the entire fermentation process. In the early stage of fermentation, *Aspergillus* was the absolute dominant genus. Although its abundance declined in the late stage of fermentation, it continued to be a major part of the flora. Additionally, *Rhizopus* is the dominant genus in tempeh [26]. *Rhizopus* and *Lichtheimia* were present, with a low abundance of 5.36% and 3.54%, respectively.

In addition to molds, yeasts widely and diversely existed in the whole fermentation system. On D0, *Apiotrichum* was the most highly abundant (11.24%) yeast, but its abundance decreased with the fermentation process. Notably, the abundance of *Millerozyma* increased significantly at the 20th day of late fermentation, reaching 89.32%, and it became the dominant strain in the late stages of Douchi fermentation. *Millerozyma farinosa*, which is common in the genus *Millerozyma*, was reported to exhibit lipolytic activity in fermented sausages, contributing to an increased level of esters and alcohols in products [27]. In addition, *M. farinosa CS2.23* was found to show excellent ester-producing ability in soy sauce [28]. Therefore, yeast may play a crucial role in the formation of esters in Douchi’s late-stage of fermentation. *Meyerozyma* was also detected in the late fermentation stage of Douchi, but its abundance was only 1.54% on the 35th day. *Meyerozyma guilliermondii*, a common yeast in the genus *Meyerozyma*, is one of the primary yeasts in Japanese and Chinese fermented soybean mash and koji [29]. It also exists in Thai soy sauce fermentation and can increase the production of volatile flavor compounds [30]. Therefore, although the abundance of *Meyerozyma* was not high in Douchi, it may contribute to some flavor attributes that were like soy sauce. In addition to the above yeasts, several salt-tolerant strains, i.e., *Candida*, *Trichosporon*, *Thermoascus*, *Kodamaea*, and *Saccharomycopsis*, were identified at the end of the fermentation process.

In summary, the fungal community showed significant compositional variation during the whole fermentation, in which mold and yeast were the two most important genera in the whole system. In the pre-fermentation stage of koji making, molds were dominant, and the total abundance was 70%. In the post-fermentation stage, yeasts rapidly gained abundance, reaching a total of nearly 90%. In the last stages of fermentation, the abundance of mold and yeast was almost equal. 

### 3.4. Beta Diversity of Aspergillus-Type Douchi during Fermentation Time

**Figure 4:**
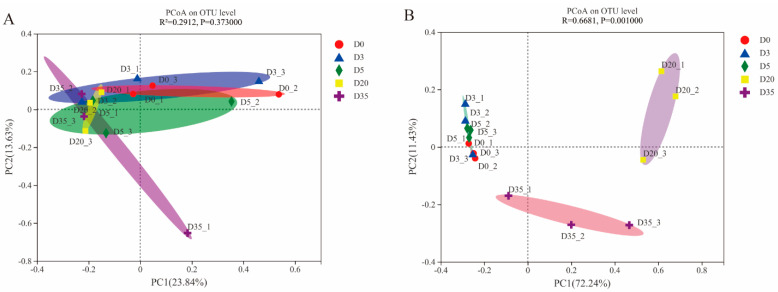
Dynamics of the microbial community in *Aspergillus*-type Douchi in different fermentation stages. Results of principal coordinate analysis (PCoA) of bacterial (**A**) and fungal (**B**) communities based on the Bray–Curtis distance matrix.

The microbial community of Douchi was clustered using a principal coordinate analysis (PCoA) approach to better understand the similarities and differences between the communities throughout each stage of fermentation. As shown in Figure 4, the distribution of the structure and composition of the bacterial community was very similar at both the pre-fermentation and post-fermentation stages, and the difference was not significant (Figure 4A). Therefore, the entire bacterial community was relatively stable during the fermentation process, and it did not show significant replacement or evolution due to environmental changes during the fermentation process. However, different from bacterial flora, the structural composition of fungal flora showed a slight difference in the primary fermentation stage but a significant difference during the secondary fermentation stage, and the two characteristic values were 72.24% (PC1) and 11.43% (PC2), respectively (Figure 4B). Combined with the data from Figure 3, it can be observed that during the production process of Douchi, the succession of fungal flora contributed to multi-level fermentation. The whole fermentation process showed the characteristics of bacteria in the whole space, mold, yeast in the time sequence, and co-fermentation of yeast and mold.

### 3.5. Dynamic Variations in Flavor Components during Douchi Fermentation

**Figure 5:**
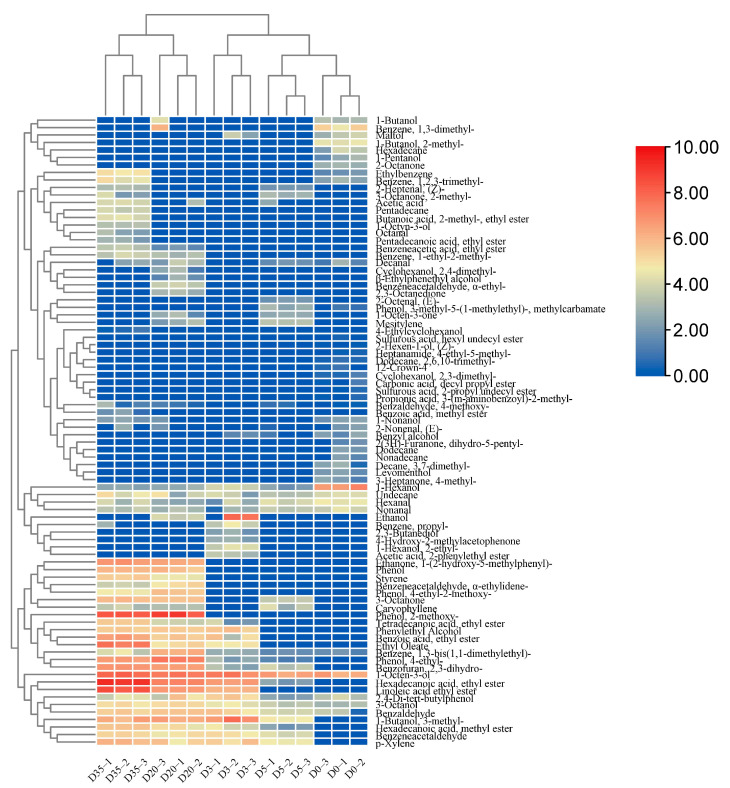
Heatmap of volatiles in Douchi based on GC–MS at different fermentation stages.

Flavor is an important characteristic of Douchi as a condiment. The flavor of fermented Douchi samples was analyzed using HS-SPME-GC-MS at five stages of fermentation. During the whole fermentation process, 83 different volatile aroma components were identified, including 20 alcohols, 12 aldehydes, 8 ketones, 7 alkanes, 2 alkenes, 15 esters, 8 benzene derivatives, 1 acid, 6 phenols, 2 furans (ketones), and 2 heterocyclic compounds (Table S2). Further analysis showed that 38 volatile components were detected on day D0, and the number increased to 48 on day D35, accompanied by an increased abundance of flavor substances (Figure 5). The analysis of the aroma threshold (odor active value, OAV) of the above volatile substances showed 17 key flavor substances in Douchi (Tables S2 and S3), which had a significant impact on the quality of Douchi, although their contents were not the highest in the overall flavor system [31].

Figure 5 shows the changes in flavor in the whole fermentation process. On day 0 (D0), the flavor substances identified were those derived from the cooked soybeans [14], mainly including 1-hexanol (118.11 ± 32.38 μg/kg), 1-octen-3-ol (84.46 ± 22.9 μg/kg), and nonanal (15.12 ± 5.14 μg/kg). During fermentation, the concentration of 1-octen-3-ol increased to 170.62 ± 42.54 μg/kg on D3, whereas benzene, 1,3-dimethyl-, 1-butanol, and 2-methyl- disappeared completely, and the contents of other flavor substances remained unchanged or decreased slightly. As fermentation proceeded, new flavor components continuously emerged. For instance, during the pre-fermentation period (D3 and D5), phenylethyl alcohol (48.39 ± 10.12 μg/kg), benzoic acid, ethyl ester (31.08 ± 23.01 μg/kg), and benzeneacetaldehyde (30.76 ± 5.45 μg/kg) were the primary newly detected flavor compounds. In addition, a large number of volatile compounds with OAV < 1 were produced, such as ethanol, 1-butanol, 3-methyl-, hexadecanoic acid, and ethyl ester.

The results in Figure 5 further showed that some new flavor substances were generated in the post-fermentation stage, and the contents of original flavor substances continued to increase, decrease, or disappear. The newly produced key flavor substances were mainly phenols and esters, such as phenol, 2-methoxy (429.98 ± 126.67 μg/kg), and phenol benzoic acid ethyl ester (55.19 ± 13.23 μg/kg). The contents of 1-octen-3-ol, hexadecanoic acid, ethyl ester, and linoleic acid ethyl ester increased continuously during the whole fermentation period. By contrast, phenol, 4-ethyl-benzofuran, and 2,3-dihydro-3-octanone were only detected in a small amount during the initial fermentation stage but also increased rapidly during the secondary fermentation stage. The contents of the above three substances increased to 177.16 ± 19.55, 94.51 ± 1.79, and 50.88 ± 0.53 μg/kg, respectively. 3-octanone with an OAV value of 45.42 had a significant effect on the characteristic flavor in the late stage. Nonanal and phenylethyl alcohol remained nearly stable until the end of fermentation; 3-methyl-1-butanol gradually decreased until it became stable; and ethanol, which was abundant in the primary fermentation stage, decreased rapidly, and eventually disappeared.

According to Table S2, the flavor distribution of Douchi showed little variation at the post-fermentation stages (D20 and D25). The main flavor components of Douchi were identified as esters, phenols, and ketones, such as butanoic acid, benzoic acid, ethyl ester, and 2-methoxy phenol. Butanoic acid, 2-methyl-, and ethyl ester (fruity flavor), which was produced at the late stage of fermentation, contributed significantly to the formation of Douchi flavor. For example, the content of butanoic acid, 2-methyl-, and ethyl ester was 17.59 ± 3.16 μg/kg, and the OAV value was up to 1353.08. Hexadecanoic acid and ethyl ester showed the highest concentration (595.47 ± 63.39 μg/kg) among all the volatile compounds identified throughout the whole fermentation stage, and it is a low-OAV (0.30) compound. In addition, some esters, alcohols, aldehydes, and acids (acetic acid) with low OAV values were detected at the end of fermentation [32].

**Figure 6:**
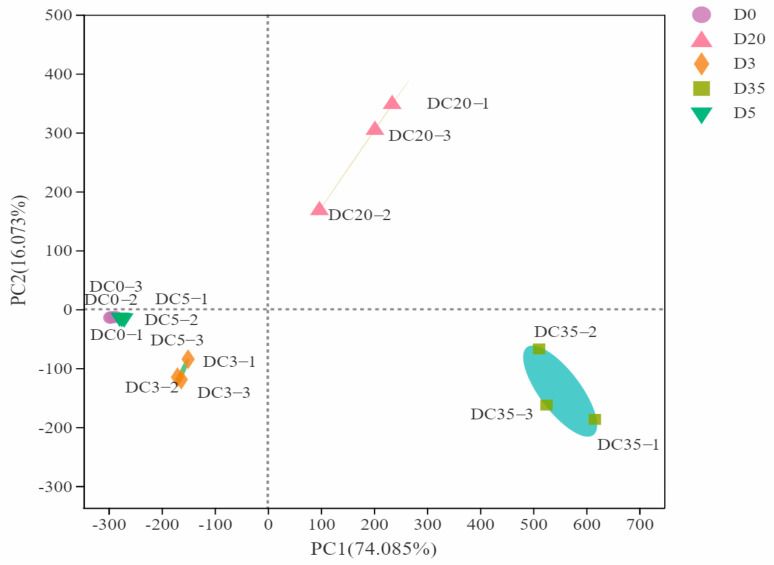
PCA plot of Douchi samples at different fermentation stages. The numbers 1, 2, and 3 represent three parallel samples.

The volatile compound concentrations were analyzed using principal component analysis (PCA) at each fermentation stage, with the results shown in Figure 6. The cumulative variance contribution of the first principal component PC1 (16.073%) and the second principal component PC2 (74.085%) was 90.158%. During the pre-fermentation stage (D0, D3, and D5), the flavor profiles showed certain similarity, and the overall flavor did not show significant difference. In the late fermentation stage (D20 and D35), as the quantity and concentration of flavor compounds increase, the overall flavor composition showed a clear distinction from that of the pre-fermentation periods, and the distinctive flavor was formed during the late fermenting period. 

In the early stage of fermentation, alcohols and aldehydes, such as 1-hexanol, 1-octen-3-ol, and nonanal, were the main compounds, which were derived from cooked soybeans. With the progress of fermentation, the relative levels of aldehydes remained stable, whereas some alcohols showed decreased concentration or disappeared. Instead, esters, ketones, and phenols, such as butanoic acid, phenol, 2-methoxy-, and 3-octanone, accumulated to become the key aroma compounds during the post-fermentation stage. The fact that alcohols were decreasing while esters were increasing in Douchi indicated that the esters were likely generated by the enzymatic interaction between higher alcohols and acetyl-CoA, mediated by ethanol acetyltransferase [33]. The reason that phenolics were able to maintain a steady growth in the late fermentation stage might be the secretion of laccase by *Aspergillus* to degrade the cell wall and release phenolic compounds [34]. In addition, hexanol, hexanal, and 1-octen-3-ol are typical bean flavor compounds [35], which were detected during all the fermentation stages. Particularly, 1-octen-3-ol shows mushroom flavor, which is the main reason for the beany smell [36]. It is known to relate to fungi and exhibited steady growth, reaching up to 251.96 ± 34.35 μg/kg in the late fermentation stage. The above results indicated that during the fermentation process, the characteristic flavor substances of the cooked beans were partially retained. New flavor compounds emerged, and some soy-derived flavor compounds (such as 1-octen-3-ol) were further accumulated due to the bacterial metabolism. 

### 3.6. Analysis of the Correlation between the Core Microbial Communities and Volatile Flavor Compounds in Douchi

**Figure 7:**
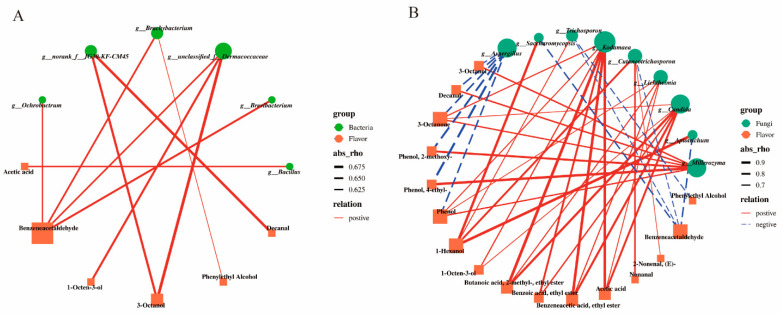
Correlation network between microbial communities and flavor compounds in Douchi during fermentation. (**A**): bacterial communities; (**B**): fungal communities. Abs_rho represents line thickness.

To further clarify the relationships between the formation of key characteristic flavors of Douchi and the core microbial community, the relative abundance data of the top 30 bacterial genera (15) and fungal genera (15) at the genus level were selected to conduct a Spearman correlation analysis of microbial communities and concentrations of 17 key flavor compounds. As shown in Figure 7, taking OAV > 1, correlation coefficient |R| ≥ 0.6, and *p* < 0.05 as the screening requirements, the results showed that six bacterial genera and nine fungal genera were associated with the characteristic flavor compounds. That was to say, the key functional microorganisms for promoting the formation of Douchi flavor were six bacterial genera and nine genera of fungi.

As depicted in Figure 7, the correlation coefficients (|R|) between bacteria and flavor substances ranged from 0.625 to 0.675, whereas those between fungi and flavor substances ranged from 0.7 to 0.9. Thus, the correlation between fungi and flavor was stronger than that between bacteria and flavor. Figure 7A shows that the flavor compounds significantly related to bacteria were mainly alcohols and aldehydes, such as phenylacetaldehyde, 1-octen-3-ol, and phenylethyl alcohol. Phenylacetaldehyde was positively correlated with *g_Ochrobactrum*, *g_Brevibacterium*, unclassified_f_Dermacoccaceae, and *g_Brachybacterium*. The unclassified_f_Dermacoccaceae was significantly associated with phenylacetaldehyde, 1-octen-3-ol, and 3-octanol. Furthermore, *Bacillus*, the most abundant microbe at the end of fermentation, correlated positively with acetic acid, the only acid compound in Douchi. *Bacillus* was the main population in fermented soybean products and had strong correlation with flavor substances [37]. Previous studies have shown that *Bacillus* has a positive correlation with the content of butyl butyrate, 1-octen-3-ol and benzaldehyde [38].

On the other hand, as demonstrated by the relationships between fungal genera and flavor compounds in Figure 7B, fungi were associated with almost all the key flavor compounds (16 species). *Aspergillus* spp., which was predominant in the pre-fermentation period, was negatively correlated with the six key flavor compounds, namely, 3-octanol, decanal, 3-octanone, 2-methoxy-phenol, 4-ethyl-phenol, and phenol. These six substances were positively correlated with *Millerozyma* spp., which was dominant in post-fermentation, indicating that the growth of and decline in *Aspergillus* spp. and *Millerozyma* spp. performed a crucial balancing function in the formation and development of Douchi flavor. In addition, *Kodamaea* spp. was positively correlated with 3-octanone, phenol, 1-octen-3-ol, 2-methyl-ethyl butyrate, ethyl benzoate, ethyl phenylacetate, and acetic acid, and *Candida* spp. was positively correlated with six flavor substances. The esters (ethyl 2-methyl-butyrate, ethyl phenylacetate, and ethyl benzoate), 3-octanone, 1-octen-3-ol, and acetic acid, which contributed considerably to the flavor of Douchi, were strongly positively linked with *Candida* spp. and *Kodamaea* spp. (*p* < 0.001), indicating that the polymorphic yeasts had a significant effect on the key characteristic flavor of Douchi. Yeast could improve the flavor of fermented soybean products, and *Candida* enhanced the sweet and caramel-like aroma of soy sauce [39]. Table S2 shows that phenols and ketones appeared in large quantities in the late stage of fermentation; their low content or absence in the early stage was probably due to the existence of mold, which inhibited their production. When the content of mold decreased in the late stage, *Millerozyma* spp. increased, and these substances were produced in large quantities and showed an important impact on flavor. The results showed that the existence of various yeasts had a vital role in the formation of various key flavor compounds in the late stage of Douchi fermentation.

## 4. Conclusions

Yangjiang Douchi is a representative of traditional fermented soybean in China. Due to factors such as manual control and natural environment change, the composition of the microbial community is different in different periods. Therefore, the quality of naturally fermented Douchi is constantly changing due to the influence of microorganisms. The purpose of this study was to clarify the relationship between the succession of microbial flora structure and the flavor characteristics of Douchi during the fermentation process through genomics and flavor composition analysis. During the fermentation of Douchi, the core functional microbial community accumulated various flavor compounds, such as 1-hexanol, 1-octene-3-ol, 3-octone, etc. However, the flavor formation of Douchi is affected by the core functional microorganisms. We focused on improving the fermentation efficiency and producing value-added products without damaging the sensory experience. We have a deep understanding of the whole fermentation process, especially the succession of microbiota, flavor development and correlation, and found out the key microbial strains that determine the aroma of Douchi. This work will make it possible to improve the quality of lobster sauce, shorten the production cycle, and improve fermentation efficiency by enriching the number of key microorganisms.

## Data Availability

Other data are contained within the Supplementary Materials.

## Supplementary Materials

The following supporting information can be downloaded at: https://www.mdpi.com/article/10.3390/foods12020329/s1, Table S1: Difference in alpha diversity of microbial community during Yangfan Douchi fermentation; Table S2: Quantitative comparison of volatile compounds (expressed in μg kg^−1^) in Yangfan Douchi during the fermentation process; Table S3: Aroma-active compounds identified by OAVs in Yangfan Douchi samples.
