# Supplementary material for: Succession and Diversity of Microbial Flora during the Fermentation of Douchi and Their Effects on the Formation of Characteristic Aroma
Source: Foods. 2023 Jan 10;12(2):329. doi: 10.3390/foods12020329 (PMC9857697; doi:10.3390/foods12020329)
Supplement: Supplementary file 1 [file foods-12-00329-s001.zip › Table S1.pdf]

foods

**Table S1.** Difference in alpha diversity of microbial community during Yangfan *Douchi* fermentation

| Sample | Diversity index | | | | Abundance index | | | | | |
|---|---|---|---|---|---|---|---|---|---|---|
| | Shannon | | Simpson | | Chao1 | | Ace | | Coverage | |
| | Bacteria | Fungi | Bacteria | Fungi | Bacteria | Fungi | Bacteria | Fungi | Bacteria | Fungi |
| D0-1 | 2.23 | 1.21 | 0.21 | 0.59 | 422.40 | 70.50 | 459.38 | 70.97 | 99.84% | 99.99% |
| D0-2 | 2.15 | 1.77 | 0.52 | 0.37 | 1271.55 | 64.14 | 1281.96 | 65.32 | 99.90% | 99.99% |
| D0-3 | 0.79 | 1.72 | 0.76 | 0.43 | 522.22 | 85.00 | 667.79 | 81.74 | 99.77% | 99.99% |
| D3-1 | 3.03 | 0.00 | 0.13 | 1.00 | 705.78 | 6.33 | 712.14 | 8.00 | 99.71% | 99.99% |
| D3-2 | 2.74 | 0.41 | 0.14 | 0.81 | 465.02 | 10.00 | 474.31 | 12.75 | 99.87% | 99.99% |
| D3-3 | 1.29 | 0.36 | 0.65 | 0.80 | 852.87 | 13.00 | 862.53 | 15.44 | 99.80% | 99.99% |
| D5-1 | 3.00 | 0.88 | 0.09 | 0.72 | 573.16 | 68.00 | 593.76 | 68.00 | 99.80% | 100.00% |
| D5-2 | 0.31 | 0.52 | 0.92 | 0.82 | 278.85 | 71.20 | 270.28 | 69.89 | 99.89% | 99.99% |
| D5-3 | 1.83 | 0.93 | 0.29 | 0.51 | 363.73 | 88.43 | 374.00 | 88.56 | 99.84% | 99.99% |
| D20-1 | 3.09 | 0.31 | 0.09 | 0.88 | 421.87 | 44.20 | 415.54 | 44.73 | 99.91% | 99.99% |
| D20-2 | 2.91 | 0.19 | 0.12 | 0.94 | 405.65 | 41.00 | 410.43 | 41.71 | 99.90% | 99.98% |
| D20-3 | 1.97 | 0.75 | 0.26 | 0.58 | 312.90 | 37.00 | 402.92 | 36.85 | 99.83% | 99.97% |
| D35-1 | 0.60 | 1.15 | 0.71 | 0.59 | 109.43 | 75.50 | 200.56 | 72.57 | 99.91% | 99.99% |
| D35-2 | 3.06 | 1.99 | 0.10 | 0.24 | 417.33 | 59.00 | 418.98 | 59.00 | 99.87% | 100.00% |
| D35-3 | 2.57 | 2.21 | 0.14 | 0.21 | 336.68 | 64.00 | 353.40 | 62.97 | 99.87% | 99.99% |
