# Supplementary material for: Succession and Diversity of Microbial Flora during the Fermentation of Douchi and Their Effects on the Formation of Characteristic Aroma
Source: Foods. 2023 Jan 10;12(2):329. doi: 10.3390/foods12020329 (PMC9857697; doi:10.3390/foods12020329)
Supplement: Supplementary file 1 [file foods-12-00329-s001.zip › Table S2.pdf]

Table S2 Quantitative comparison of volatile compounds (expressed in μg kg^{-1}) in Yangfan *Douchi* during the fermentation process.

No.	Volatile compound	Content (μg kg-1)					RI	LRI	Identification
		D0	D3	D5	D20	D35			
**Alcohols**									
1	Ethanol	ND	226.37±31.42a	ND	13.78±2.74b	ND	427	812	MS, RI, LRI
2	1-Butanol	8.09±1.18a	ND	ND	6.25±10.82a	ND	659	1062	MS, RI, LRI
3	1-Butanol, 3-methyl-	ND	152.97±68.48a	25.01±1.9b	84.01±14.34b	77.49±22.38b	736	1137	MS, RI, LRI
4	Levomenthol	3.1±1.03	ND	ND	ND	ND	1175	1140	MS, RI, LRI
5	1-Butanol, 2-methyl-	20.08±1.94	ND	ND	ND	ND	739	1127	MS, RI, LRI
6	1-Pentanol	4.71±2.73	ND	ND	ND	ND	765	1172	MS, RI, LRI
7	Cyclohexanol, 2,4-dimethyl-	ND	ND	ND	4.46±3.35	ND	1032	1192	MS, RI, LRI
8	1-Octen-3-ol	84.46±22.9c	170.62±42.54b	90.57±14.39c	188.99±18.09b	251.96±34.35a	980	1386	MS, RI, LRI
9	Cyclohexanol, 2,3-dimethyl-	0.77±0.53	ND	ND	ND	ND	-	1418	MS, RI, LRI
10	4-Ethylcyclohexanol	ND	ND	ND	ND	0.55±0.4	1003	1440	MS, RI, LRI
11	1-Hexanol, 2-ethyl-	ND	15.95±4.49	ND	ND	ND	1030	1454	MS, RI, LRI
12	1-Octyn-3-ol	ND	ND	ND	ND	8.93±0.54	-	1216	MS, RI, LRI
13	1-Hexanol	118.11±32.38a	14.44±2.23b	3.41±1.1b	5.46±0.93b	4.5±0.58b	868	1289	MS, RI, LRI
14	2-Hexen-1-ol, (Z)-	0.06±0.06	ND	ND	ND	ND	868	1561	MS, RI, LRI
15	2,3-Butanediol	ND	4.12±1.94	ND	ND	ND	788	1560	MS, RI, LRI
16	3-Octanol	7.4±1.29c	21.43±3.64b	10.91±1.95c	35.26±2.54a	31.78±4.4a	994	1339	MS, RI, LRI
17	1-Nonanol	3.57±1.1a	ND	ND	0.97±1.68b	3.59±0.93a	1173	1539	MS, RI, LRI
18	Benzyl alcohol	3.46±1.52a	1.3±1.14b	0.36±0.63b	ND	ND	1036	1970	MS, RI, LRI

19	Phenylethyl Alcohol	ND	48.39±10.12a	ND	44.16±1.93a	43.66±3.57a	1116	2024	MS, RI, LRI
20	β-Ethylphenethyl alcohol	ND	ND	ND	3.47±2.7	ND	1261	2123	MS, RI, LRI
**Aldehydes**									
21	Octanal	ND	ND	ND	ND	5.49±2.91	1003	1199	MS, RI, LRI
22	2-Heptenal, (Z)-	ND	ND	2.82±0.12^{b}	ND	7.98±0.57^{a}	958	1236	MS, RI, LRI
23	Hexanal	22.14±3.13^{a}	7.53±7.12^{bc}	15.44±3.83^{ab}	4.7±0.54^{c}	12.4±5.4^{bc}	800	927	MS, RI, LRI
24	Decanal	3.12±3.11^{a}	ND	2.44±0.41^{a}	7.47±4.35^{a}	2.99±2.64^{a}	1206	1453	MS, RI, LRI
25	Nonanal	15.12±5.14^{a}	4.31±3.41^{c}	10.5±1.38^{ab}	7.39±1.91^{bc}	9.96±2.25^{abc}	1104	1323	MS, RI, LRI
26	Benzaldehyde	7.44±6.16^{b}	39.38±13.56^{a}	14.76±1.23^{b}	49.52±1.99^{a}	41.16±6.4^{a}	962	1465	MS, RI, LRI
27	2-Nonenal, (E)-	2.26±2.01^{a}	ND	0.12±0.21^{a}	0.96±1.66^{a}	1.87±3.25^{a}	1162	1493	MS, RI, LRI
28	2-Octenal, (E)-	ND	ND	2.9±0.51	ND	ND	1060	1358	MS, RI, LRI
29	Benzeneacetaldehyde	ND	30.76±5.45^{ab}	15.97±2.44^{c}	28.08±6.18^{b}	37.85±2.76^{a}	1045	1619	MS, RI, LRI
30	Benzeneacetaldehyde, α-ethyl-	ND	ND	ND	12.01±1.87	ND	-	1693	MS, RI, LRI
31	Benzaldehyde, 4-methoxy-	0.12±0.11^{a}	ND	0.43±0.38^{a}	0.88±0.48^{a}	3.19±3.84^{a}	1250	2164	MS, RI, LRI
32	Benzeneacetaldehyde, α-ethylidene-	ND	ND	ND	32.61±6.47^{a}	12.55±1.33^{b}	1279	2035	MS, RI, LRI
**Ketones**									
33	3-Heptanone, 4-methyl-	3.14±1.67	ND	ND	ND	ND	-	827	MS, RI, LRI
34	3-Octanone	ND	ND	11.02±0.03^{c}	50.88±0.53^{b}	59.04±5.99^{a}	986	1156	MS, RI, LRI
35	2-Octanone	6.17±0.59	ND	ND	ND	ND	990	1201	MS, RI, LRI
36	1-Octen-3-one	ND	ND	5.02±0.36^{a}	5.11±3.28^{a}	ND	979	1220	MS, RI, LRI
37	2,3-Octanedione	ND	ND	ND	7.04±1.8	ND	984	1240	MS, RI, LRI
38	3-Octanone, 2-methyl-	ND	ND	6.55±1.25^{ab}	ND	7.82±7.7^{a}	985	1243	MS, RI, LRI
39	Ethanone, 1-(2-hydroxy-5-methylphenyl)-	ND	ND	ND	80.63±7.07^{b}	109.58±14.59^{a}	1316	2390	MS, RI, LRI
40	4-Hydroxy-2-methylaceto-phenone	ND	4.88±1.45	ND	ND	ND	-	2395	MS, RI, LRI

**Alkenes**									
41	Undecane	16.58±1.24[a]	10.81±6.74[a]	8.97±0.85[a]	15.67±12.74[a]	23.73±9.51[a]	1100	1001	MS, RI, LRI
42	Dodecane	2.27±2.07	ND	ND	ND	ND	1200	1112	MS, RI, LRI
43	Hexadecane	9.54±5.88	ND	ND	ND	ND	1600	1163	MS, RI, LRI
44	Decane, 3,7-dimethyl-	3.56±2.89	ND	ND	ND	ND	1125	1220	MS, RI, LRI
45	Dodecane, 2,6,10-trimethyl-	0.46±0.19	ND	ND	ND	ND	1366	1385	MS, RI, LRI
46	Nonadecane	1.87±1.97	ND	ND	ND	ND	1900	1393	MS, RI, LRI
47	Pentadecane	ND	ND	ND	ND	13.14±2.38	1500	1459	MS, RI, LRI
**Olefin**									
48	Styrene	ND	ND	ND	21.65±0.9[b]	43.3±2.83[a]	893	1150	MS, RI, LRI
49	Caryophyllene	ND	ND	11.55±6.37[a]	8.68±0.39[a]	10.94±3.71[a]	1419	1556	MS, RI, LRI
**Esters**									
50	Butanoic acid, 2-methyl-, ethyl ester	ND	ND	ND	ND	17.59±3.16	849	882	MS, RI, LRI
51	Carbonic acid, decyl propyl ester	0.46±0.66	ND	ND	ND	ND	-	1244	MS, RI, LRI
52	Sulfurous acid, hexyl undecyl ester	0.11±0.17	ND	ND	ND	ND	-	1314	MS, RI, LRI
53	Benzoic acid, methyl ester	ND	ND	ND	ND	2.45±1.84	1171	1599	MS, RI, LRI
54	Tetradecanoic acid, ethyl ester	ND	6.74±7.44[c]	ND	13.3±1.57[b]	44.7±0.59[a]	1794	2229	MS, RI, LRI
55	Pentadecanoic acid, ethyl ester	ND	ND	ND	ND	5.17±1.92	1894	2364	MS, RI, LRI
56	Sulfurous acid, 2-propyl undecyl ester	0.18±0.25	ND	ND	ND	ND	-	1648	MS, RI, LRI
57	Benzoic acid, ethyl ester	ND	31.08±23.01[b]	ND	43.47±6.4[b]	93.18±14.96[a]	1171	1656	MS, RI, LRI
58	Benzeneacetic acid, ethyl	ND	ND	ND	2.34±0.75[b]	10.38±1.83[a]	1246	1822	MS, RI, LRI

	ester								
59	Acetic acid, 2-phenylethyl ester	ND	9.08±2.44	ND	ND	ND	1258	1870	MS, RI, LRI
60	Phenol, 3-methyl-5-(1-methylethyl)-, methylcarbamate	0.58±0.53[b]	ND	5.66±1.68[a]	ND	ND	1687	2393	MS, RI, LRI
61	Hexadecanoic acid, methyl ester	ND	18.19±7.16[c]	3.38±0.95[d]	28.99±7.84[b]	50.46±1.29[a]	1926	2439	MS, RI, LRI
62	Hexadecanoic acid, ethyl ester	ND	98.56±5.33[c]	3.74±1.07[d]	152.58±5.34[b]	595.47±63.39[a]	1993	2493	MS, RI, LRI
63	Linoleic acid ethyl ester	ND	66.05±1.2[b]	ND	102.73±1.41[b]	345.39±49.75[a]	2162	2860	MS, RI, LRI
64	Ethyl Oleate	ND	26.42±2.31[b]	ND	34.09±6.87[b]	189.39±24.39[a]	2173	2780	MS, RI, LRI
**Benzene derivative**									
65	Ethylbenzene	2.44±0.67[b]	ND	ND	ND	29.04±3.03[a]	855	1024	MS, RI, LRI
66	p-Xylene	ND	42.45±13.19[b]	23.27±1.08[c]	39.41±14.82[bc]	63.59±8.41[a]	865	1033	MS, RI, LRI
67	Benzene, 1,3-dimethyl-	35.08±10.97[a]	ND	ND	23.01±39.86[a]	ND	866	1038	MS, RI, LRI
68	Benzene, 1-ethyl-2-methyl-	ND	ND	ND	8.14±1.67[b]	12.79±3.11[a]	970	1105	MS, RI, LRI
69	Mesitylene	ND	ND	9.33±0.42[a]	5.69±2.79[b]	ND	972	1175	MS, RI, LRI
70	Benzene, 1,2,3-trimethyl-	4.9±1.65[b]	ND	ND	ND	24.18±8.48[a]	1013	1185	MS, RI, LRI
71	Benzene, 1,3-bis(1,1-dimethylethyl)-	2.25±0.77[c]	5.39±0.39[c]	2.18±0.36[c]	73.33±5.35[a]	16.82±6.72[b]	1247	1361	MS, RI, LRI
72	Benzene, propyl-	ND	15.58±8.01	ND	ND	2.13±3.68	953	1633	MS, RI, LRI
**Acids**									
73	Acetic acid	ND	ND	1.64±2.83[b]	2.72±4.71[b]	15.61±1.27[a]	610	1392	MS, RI, LRI
**Phenols**									
74	Phenol, 2-methoxy-	ND	ND	ND	429.98±126.67[a]	324.66±25.75[b]	1090	1935	MS, RI, LRI
75	Phenol, 4-ethyl-	ND	5.4±2.61[c]	1.41±0.31[c]	177.16±19.55[a]	98.96±8.04[b]	1169	2370	MS, RI, LRI
76	Phenol	ND	ND	ND	55.19±13.23[a]	62.93±2.49[a]	980	2150	MS, RI, LRI

77	Phenol, 4-ethyl-2-methoxy-	ND	ND	ND	49.96±5.49[a]	24.79±2.29[b]	1282	2181	MS, RI, LRI
78	2,4-Di-tert-butylphenol	11.07±3.75[bc]	40.12±9.91[a]	3.74±0.72[c]	30.63±22.03[ab]	17.39±4.81[bc]	1519	2546	MS, RI, LRI
79	Maltol	10.32±4.18[a]	5.53±6.38[ab]	ND	ND	ND	1110	2096	MS, RI, LRI
**Furan(one)s**									
80	2(3H)-Furanone, dihydro-5-pentyl-	1.42±1.3	ND	ND	ND	ND	1363	2178	MS, RI, LRI
81	Benzofuran, 2,3-dihydro-	ND	6.19±3.24[c]	11.48±3.56[c]	94.51±1.79[b]	112.54±4.26[a]	1224	2629	MS, RI, LRI
**Others**									
82	Heptanamide, 4-ethyl-5-methyl-	0.16±0.15	ND	ND	ND	ND	-	1824	MS, RI, LRI
83	12-Crown-4	0.43±0.43	ND	ND	ND	ND	-	2787	MS, RI, LRI

ND: not detected.

Calculated retention index (RI) on DB-WAX column.

Identification methods: MS, mass spectrometry; RI, retention indices; LRI, linear retention indices.
