# Supplementary material for: Succession and Diversity of Microbial Flora during the Fermentation of Douchi and Their Effects on the Formation of Characteristic Aroma
Source: Foods. 2023 Jan 10;12(2):329. doi: 10.3390/foods12020329 (PMC9857697; doi:10.3390/foods12020329)
Supplement: Supplementary file 1 [file foods-12-00329-s001.zip › Table S3.pdf]

foods

MDPI

Table S3. Aroma-active compounds identified by OAVs in Yangfan *Douchi* samples.

No.	Volatile compound	Threshold value (mg kg^{-1})	OAV	Smell characteristics
8	1-Octen-3-ol	0.01	25.2	Mushroom fragrance
13	1-Hexanol	0.034	3.47	Beany, fruity
16	3-Octanol	0.018	1.77	earthy，mushroom，musty，fermented
19	Phenylethyl Alcohol	0.012-0.021	2.08-3.64	Fragrance, floral
24	Decanal	0.0001	29.9	floral
25	Nonanal	0.0031	4.88	Flower, orange, grassy
27	2-Nonenal, (E)-	0.00019	9.842	fatty
29	Benzeneacetaldehyde	0.0063	6.01	Fermented, earthy
34	3-Octanone	0.0013	45.42	Mildew, fragrance, mushrooms
36	1-Octen-3-one	0.00012	42.583	Mushroom
50	Butanoic acid, 2-methyl-, ethyl ester	0.000013	1353.08	Fruity
57	Benzoic acid, ethyl ester	0.053	1.76	fruity, medicinal
58	Benzeneacetic acid, ethyl ester	0.0033	3.15	balsam，cocoa
73	Acetic acid	0.013-0.15	1.2	Sour, pungent
74	Phenol, 2-methoxy-	0.0025	17.2	Smoky
75	Phenol, 4-ethyl-	0.042	4.22	Smoky, savory
76	Phenol	0.021	3	Rubbery, plastic-like
